# Spontaneous Tonsillar Hemorrhage due to Infectious Mononucleosis

**DOI:** 10.7759/cureus.10367

**Published:** 2020-09-10

**Authors:** Andrew Wahba, Rafik ElBeblawy

**Affiliations:** 1 Pediatrics, University of Texas Health Science Center at Houston McGovern Medical School, Houston, USA; 2 Infectious Diseases, University of Louisville School of Medicine, Louisville, USA

**Keywords:** spontaneous tonsillar hemorrhage, hematemesis, tonsillectomy, infectious mononucleosis

## Abstract

Spontaneous tonsillar hemorrhage is defined as continuous bleeding for more than one hour, or more than 250 mL of blood loss regardless of the duration of bleeding. It is associated with various pathologic conditions, including acute or chronic tonsillitis, peritonsillar or parapharyngeal abscess, infectious mononucleosis, carotid aneurysm or pseudoaneurysm, and tonsil cancer. It is a rare complication with very limited data reported in the literature. Reported cases indicate an increased incidence in young patients, associated with a higher mortality rate. We report a rare case of spontaneous tonsillar hemorrhage due to infectious mononucleosis in a previously healthy 16-year-old female.

## Introduction

Spontaneous tonsillar hemorrhage (STH) is a rare event, and is defined as continuous bleeding for more than one hour, or more than 250 mL of blood loss regardless of the duration of bleeding [1]. The overall incidence of STH is 1.2% from all susceptible pathologies [1,2]. Tonsillitis (acute or chronic) is the most common predisposing factor for more than 80% of STH cases [3-6]. It is associated with other pathologic conditions, including peritonsillar or parapharyngeal abscess, infectious mononucleosis, carotid aneurysm or pseudoaneurysm, tonsil cancer or bleeding disorders [1,3,7-9]. It is most commonly reported in the age group 10 to 20 years [3]; however, it was reported in infants [5,10] and older adults [1,11]. Prior to the antibiotic era, it was believed that the pathophysiology of this condition is due to invasion of the aberrant vessels by the inflamed tonsil or peritonsillar abscess causing significant vessel erosion [12]. Also, increased blood flow due to the inflammatory state precludes to increased susceptibility of tonsillar bleeding. Given the rarity of this presentation in clinical practice, mortality has been reported in children [13], and guidelines for immediate control and management are implemented. In 2010, a study by Salem et al. [3] reported 55 cases of STH in the medical literature in four decades, and only three of them were due to infectious mononucleosis [1,9,14].

In this report, we identify a case of STH in a pediatric patient secondary to infectious mononucleosis; clinical presentation, laboratory findings, imaging, pathology, and management are discussed.

## Case presentation

A previously healthy 16-year-old Caucasian female presented to our emergency department (ED) for hematemesis and hemoptysis. She has had three days of fever to 102˚F, sore throat, and fatigue. Over the past two days, she had three episodes of blood coming from her mouth, not preceded by coughing. She was seen at an urgent care facility and was found to be streptococcal and monospot negative. The patient's mother endorsed that the patient was initially "spitting up blood" as opposed to vomiting blood today, thus prompting her to visit the ED. At our ED, she had one episode of hematemesis of 300 mL. The patient also reported mid epigastric abdominal pain. She had not taking nonsteroidal anti-inflammatory drugs (NSAID) or any other medications. At ED, she was afebrile 98.3˚F, tachycardic, and normotensive. 

On physical examination, she had normal nasal mucosa and inferior turbinates with midline septum. Lips, teeth, gums were normal and moist oral mucosa without visible lesions. Tongue and floor of the mouth without masses, clear oropharynx, palate, and uvula without lesions and with symmetric elevations were identified. There was no palpable cervical adenopathy. No neck masses or submandibular gland tenderness was noted. 

**Figure 1:**
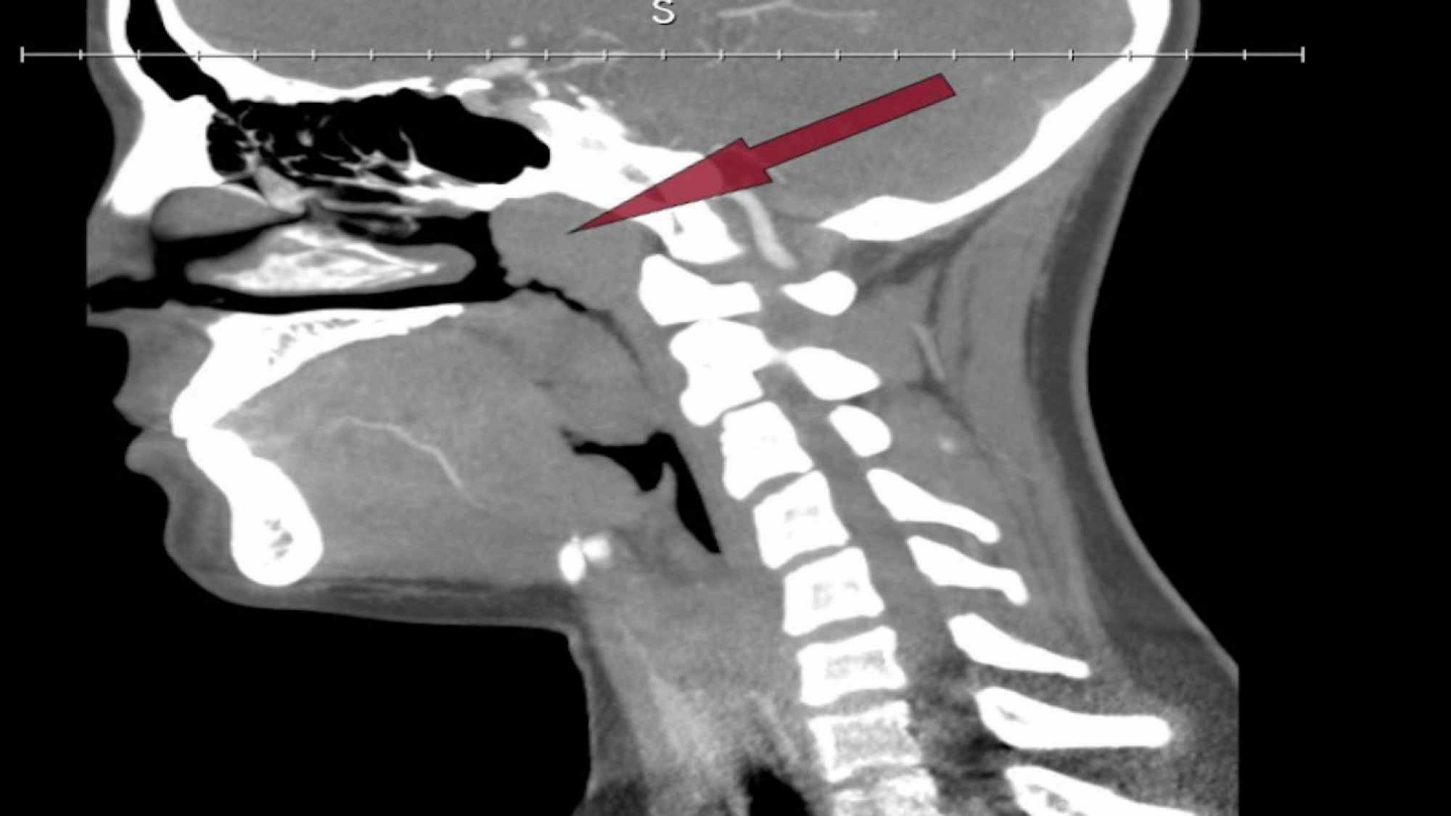
CT angiogram of the head and neck showing enlargement of the nasopharyngeal and oropharyngeal lymphoid tissue.

The initial laboratory revealed hemoglobin (Hgb) 12.2 g/dL, hematocrit (Hct) 35.7%, and platelet 204 x 10^3^/mm^3^ which dropped after three hours to 9.4, 27, and 147, respectively (Table 1). The chest radiograph (CXR) was normal. CT of head and CT angiogram of the head and neck were done that only showed enlarged nasopharyngeal lymphoid tissue (Figure 1).

 

**Table 1:** Initial laboratory, after 3 and 12 hours WBC, white blood cell; RBC, red blood cell;  MCV, mean corpuscular volume; RDW, red blood cell distribution width; INR, international normalized ratio; APTT, activated partial thromboplastin time; BUN, blood urea nitrogen; ALT, alanine aminotransferase; SGPT, serum glutamic pyruvic transaminase; AST, aspartate aminotransferase; SGOT, serum glutamic oxaloacetic transaminase; Alk Phos, alkaline phosphatase

	Initial	After 3 hours	After 12 hours
WBC, ×10^3^/mm^3^	6.8	4.9	
RBC, ×10^6^/mm^3^	4.2	3.24	
Hemoglobin, g/dL	12.2	9.4	8.5
Hematocrit, %	35.7	27.6	23.8
MCV, fL	85.2	85.2	
RDW, %	13.1	12.8	
Platelet, ×10^3^/mm^3^	204	147	
Neutrophil, %	81.7%	76.6%	
Lymphocyte, %	12.7%	8.2%	
Eosinophil, %	0.1%	0.1%	
Neutrophil, ×10^3^/mm^3^	5.2	4.4	
Monocytes, ×10^3^/mm^3^	0.7	0.5	
Lymphocyte, ×10^3^/mm^3^	0.9	0.4	
Protime, seconds	13.9		
INR	1.09		
APTT, seconds	46.9		
Sodium, mEq/L	136		
Potassium, mEq/L	3.4		
Chloride, mEq/L	103		
CO_2_ level, mEq/L	27		
Anion gap, mEq/L	9.4		
Creatinine, mg/dL	0.76		
BUN, mg/dL	19		
Calcium, mg/dL	8.9		
Phosphorus, mg/dL	3.2		
Magnesium, mg/dL	1.9		
ALT(SGPT), unit/L	23		
AST(SGOT), unit/L	15		
Alk Phos, unit/L	77		
Total bilirubin, mg/dL	0.2		
Acetaminophen level, µg/mL	<2		
Salicylate level, mg/dL	<1.7		

At ED, she received pantoprazole, a 20 cc/Kg normal saline bolus, and was admitted for further workup. Pediatric gastroenterology and pediatric otolaryngology were consulted. A flexible fiberoptic laryngoscopy was performed, and granulation tissue on the superior aspect of the right tonsil was noted but no source of hemorrhage was identified. After 12 hours, Hgb dropped to 8.5 g/dL and Hct 23.8% (Table 1). 

Because of the acute drop in Hgb and continued symptoms, she underwent emergent esophagogastroduodenoscopy. The anesthesia team performed bronchoscopy at the time of intubation, and no source of bleeding was identified.

Results of mucosal biopsy from the mid and distal esophagus and duodenum showed no significant histopathologic alterations. Mucosal biopsy from the stomach showed chronic gastritis with focal activity. Immunohistochemical stain for *Helicobacter pylori* was negative. 

Given the negative endoscopy and area of granulation tissue in the setting of sore throat, the decision was made to perform emergent tonsillectomy for suspected STG. There was complete resolution of symptoms after surgery. 

Bilateral tonsillar tissue surgical pathology and flow cytometry immunophenotyping (Table 2) showed marked follicular hyperplasia with focal ulceration, Epstein-Barr virus (EBV) positivity, and no features of lymphoma. Immunostains with appropriate controls were performed. CD2/CD3 and CD20/PAX5 showed appropriate T/B-cell distribution. Pan T-cell markers (CD2/CD3) showed that T lymphocytes were around 24% without loss of pan T-marker. CD4/CD8 ratio was 2.8. NK cells were 1% and B-cells were 76% of lymphocytes and polyclonal. The germinal centers were marked by BCL6 with a high proliferation index by Ki-67 as a normal pattern. The germinal centers were negative for BCL2. Plasma cells were increased by MUM1 stain. There were many small to medium-sized lymphoid cells marked by CD30 in paracortical areas. They were negative for CD15, MUM1, BCL6, and ALK1. EBER (EBV encoded RNA by in situ hybridization) showed scattered EBER+ lymphoid cells, suggestive of EBV infection. There was no aberrant phenotype identified by flow cytometry.

**Table 2:** Cell surface marker results ALL, acute lymphoblastic leukemia; CALLA, common acute lymphoblastic leukemia antigen

Cluster	Specificity	% gated cells
CD2	Pan-T	28
CD3	Pan-T	24
CD4	Helper-T	17
CD8	Suppressor-T	6
CD5	T, B subsets	25
CD7	T-ALL Ag	21
CD10	Common ALL (CALLA)	16
CD16	Natural killer cells	1
CD56	Natural killer cells	7
CD19	Pan-B	76
CD20	Pan-B	74
CD22	Restricted-B	78
CD23	B cells	27
FMC7	B cells	56
CD11c	Monocytic/HCL Ag	6
7AAD	Viability	83

Tonsillar pathology results showed findings consistent with reactive follicular hyperplasia. No diagnostic features of lymphoma were found. There was marked follicular hyperplasia and chronic inflammation with focal ulceration in the right tonsil along with follicular hyperplasia and focal abscesses in the left tonsil.

## Discussion

STH is a rare complication of infectious mononucleosis. The pathophysiology of increased bleeding susceptibility is the state of inflammation of the tonsils resulting in increased blood flow and engorgement. Using a xenon-133 clearance technique, increased tonsillar blood flow was detected in hypertrophic and inflamed tonsils [15]. Surface and adjacent vessels can be eroded from local tonsillar ulceration and abscess formation, as pathologically detected in our patient. In a study by Johnsen et al. in 1984, tonsillar bleeding was a complication reported in only 2 out of 467 (0.4%) patients with infectious mononucleosis [16]. Therefore, STH, though rare, should be included in the differential diagnosis of hematemesis, hemoptysis, and posterior epistaxis, similar to our patient [5]. STH presents as intermittent bleeding or blood clots identified in the mouth.

In mild cases, conservative local control with nebulized adrenaline, H_2_O_2_ gargle, antibiotic therapy, or cauterization can be used to control bleeding. However, if local control is not successful, severe STH recurs or malignancy is suspected, and therefore tonsillectomy is recommended [3,10]. Literature reports on STH are scarce and are usually confined to case reports and series. This case advances the literature by providing a rare instance of STH due to infectious mononucleosis in a pediatric patient, and highlights the clinical presentation and challenges of management.

## Conclusions

STH is a rare complication of acute and chronic tonsillitis. It can happen in infectious mononucleosis due to the inflammatory condition and increased blood flow to the tonsils. It should be included in the differential diagnoses of hematemesis, hemoptysis, and posterior epistaxis. Management includes local conservative control or tonsillectomy according to severity.
